# How Do We Get From Good to Great? The Need for Better Observation Studies of Creativity in Education

**DOI:** 10.3389/fpsyg.2018.02342

**Published:** 2018-11-27

**Authors:** Jen Katz-Buonincontro, Ross C. Anderson

**Affiliations:** ^1^School of Education, Drexel University, Philadelphia, PA, United States; ^2^Department of Educational Methodology Policy and Leadership, University of Oregon, Eugene, OR, United States

**Keywords:** creativity processes, observation, literature review, research methods education, qualitative methodology, quantitative methodology

Without quality research using observation methods, it is difficult to understand creativity processes in action, especially in the field of education. But, what is the current state of these studies? Based on a review of 37 extant studies, we found that observation is surprisingly under-utilized, and that more rigorous observation studies of creativity processes in education are needed.

Firsthand accounts of behaviors, interactions, and discussions between individuals or groups (Merriam and Tisdell, 2016) is an essential affordance of observation. In education, observation of student creativity focuses mostly on talented and gifted student identification (Plucker and Makel, 2010), with lesser attention to the classroom context and interactions among teachers and students. As most creativity studies are informed by psychological research methods and thus favor quantitative methods, often in the tradition of psychometrics and experimental design (e.g., Plucker and Renzulli, 1999; Plucker and Makel, 2010), observation methods have received relatively less attention. In this opinion article, we review 37 published studies using quantitative behavioral observation or qualitative naturalistic observation to study creativity in education, across 38 years. Our aim was to parse out the strengths and weaknesses of the articles and recommend improvements for the rigor of observation studies (see Table 1).

**Table 1:** Recommendations to address methodological weaknesses in observation of creativity in education.

**Research element**	**Quantitative weaknesses**	**Qualitative weaknesses**	**Recommendations**
Theory	Inclusion of too many theories of creativity or learning	Creativity defined in numerous ways including terms such as “imagination”	Select one creativity definition or model and use it consistently
Observation protocol	Lack of alignment between the teaching and learning context and the observation protocol design	Unspecified or general activities observed	Provide rationale for observing learning activities and how they are linked to creativity
Role of observer	Assumed objectivity in observer role	Type of observer role is unclear	Identify observer role on continuum from participant to non-participant/direct Test for the mediation and moderation effects of specific environmental conditions
Sampling strategy	Convenience sampling used as a default	No reason provided for sampling classrooms	Describe sampling rationale and method such as peer-nominated teachers
Research design	Limited to descriptive and associational designs; cross-sectional mediation analysis	Unspecified research design and type of mixed methods	Be specific with type of design Declare type of mixed methods design Consider other types of analyses to make more precise contributions to theory
Data analysis	Limited to descriptive statistics only	Observation data is blended with interview and survey results	Report observation data under separate heading in methods and results sections Use proper and rigorous analytic procedures
Validity and reliability	Little discussion of validity and reliability of observation data	Minimal discussion of variance in creativity across instances of teacher-student interactions	Employ “thick description” of naturalistic settings Triangulate observation data with other qualitative or quantitative data Aggregate instances of creative processes and plot trends.

## Contributions of quantitative behavioral observation

In quantitative observation methods, the researcher records evidence of individual behaviors or interactions (Creswell, 2008), but this method is very limited in educational creativity studies. In this review, we used search terms “observation/observational methods/or observational learning,” combined with “education,” and “creativ^*^,” “think,^*^” “problem solv^*^,” or “behav^*^” in ERIC, Education Research Complete, Art Education and PsycInfo databases, and specific journal archives. Nine articles were identified from 1980 to 2018 using quantitative observation methods for studying the learning process or environments related to creativity. Seven measures focused on environmental or instructional support for creativity in K-12 educational settings—five domain-general settings (5), one in K-12 science classrooms (1), and one in a physical education class (1). Two additional studies examined the creative process in different types of tasks (see Table 1).

In these articles, observational methods tested theories and evaluated educators. For example, Ruscio et al. (1998) recorded and measured aspects of the creative process of individuals in written, constructive, and artistic tasks. Torrents et al. (2010) used observation to measure creative movement in improvised physical interaction between two people. Schacter et al. (2006) investigated the relationship between observed teaching practices for creativity and students' subsequent academic achievement. One of the five studies published between 2016 and 2018 used observation to explore which aspects of support for creativity were most frequent in science instruction (Al-Abdali and Al-Balushi, 2016). Richardson and Mishra (2018) built their measure to help educators and school administrators improve the design of learning environments to support creativity. Konstantinidou and Zisi (2017) produced an 18-item checklist to evaluate the level of support for creativity in physical education classes. Gadja et al. (2017) observed both teachers and student behaviors separately using a checklist of exemplary creative behaviors in teaching and learning.

### Creativity framework

Most studies developed a framework of creative teaching behaviors through reviews of research on creativity and instructional practices within their domain of interest (Al-Abdali and Al-Balushi, 2016; Konstantinidou and Zisi, 2017; Richardson and Mishra, 2018). In some cases, researchers compared findings from past research with inductive findings from their own field notes in specific settings. Konstantinidou and Zisi (2017) adapted an extant self-report measure aligned to Cropley's (1997) framework to create a short behavioral observation checklist of the environment. Similarly, Furman (1998) applied the Origin-Pawn Questionnaire as a criterion for the cognitive, social, and emotional support for creativity. Al-Abdali and Al-Balushi (2016) framed their approach specifically around different science teaching approaches that support creativity. In exploratory studies, creativity theory guided a process that was largely inductive (Ruscio et al., 1998). Pitts et al. (2018) constructed an observation tool from a developmental framework for creativity in learning. Gadja et al. (2017) built their checklist of behaviors from an extant model of creativity in education.

The strongest quantitative observation approaches linked creativity theory explicitly to frameworks for instructional practices (Al-Abdali and Al-Balushi, 2016; Pitts et al., 2018; Richardson and Mishra, 2018). For example, Richardson and Mishra (2018) developed observation categories based on an extant observation protocol of school administrators, field notes in peer-nominated creative classrooms, and theory from creativity research.

### Quantitative data collection and analysis methods

Schacter et al. (2006) observed 48 elementary school teachers eight different times for a whole class period using ethnographic field notes as the basis for ratings. Some studies provided extensive detail about the procedures used to observe and rate the environment (Schacter et al., 2006; Konstantinidou and Zisi, 2017; Richardson and Mishra, 2018) and others provided much less specificity (Ruscio et al., 1998; Al-Abdali and Al-Balushi, 2016). Generally, sampling decisions were based on convenience rather than analytic power.

Scoring procedures dictated the analytic approach for the observations. Across protocol researchers seemed to keep the number of indicators to score below 20. Most studies used a general response options for rating (e.g., “no evidence” to “fully present”); only Torrents et al. (2010) and Konstantinidou and Zisi (2017) used a behavioral checklist with actual frequency counts. Most studies included more than one observation instance and each study pursued inter-coder reliability, generally using the 80% threshold of inter-observer agreement across raters. In their analyses, most studies reported the descriptive statistics for each category or domain of indicators. Some researchers explored what different levels of frequency or quality might indicate for theory and practice (Schacter et al., 2006; Torrents et al., 2010; Al-Abdali and Al-Balushi, 2016; Konstantinidou and Zisi, 2017). Gadja et al. (2017) compared observation scores between classrooms identified as having a null, positive, or negative relationship between students' creative ability and academic achievement.

Each study established initial validity and usability of the instrument to observe for support for creativity. Like Al-Abdali and Al-Balushi (2016), other studies used a panel of judges germane to the context (e.g., science educators) to evaluate the face validity of the protocol and improve its practical significance. These quantitative studies represent early, exploratory work to promulgate research about the creative process and to improve the affordances of learning environments in support of creativity.

## Contributions of qualitative observation research

Qualitative observation methods were more prevalent than quantitative and mixed methods for observing creativity in learning environments. Twenty-six articles were found in ERIC and PsycInfo databases from 1980 to 2018 using qualitative observation methods for studying creativity processes in visual art (14), music education (3), theater (1), fashion (1), science, computer science and mathematics education (5), early childhood/non-domain specific (1), and technology (1).

### Creativity framework

Definitions of creativity ranged, and some authors elected to include multiple definitions (e.g., Pitri, 2013), making it difficult to pinpoint the study's contributions to specific creativity theories. Cognitive components of divergent and convergent thinking—fluency and flexibility—were emphasized (e.g., Karademir, 2016). Robson and Rowe (2012) used a creative thinking framework that conflated “imagination” with “creativity,” explaining it as originality, novelty, and even critical thinking. In arts and music education studies, the creativity focus shifted from cognitive components to more expressive qualities such as collaboration (Biasutti, 2015), modeling and employment of art materials (James, 1997; Guay, 2000; Kandemir and Gur, 2007; Geist and Hohn, 2009; Thomas, 2009; Walker, 2014; Budge, 2016; Lorimer, 2016; Mars, 2016), creative “encounters” (Petsch, 2000), aesthetic or flow experience (Vuk et al., 2015), and transformation (Walker, 2014).

While some articles described the research focus, such as teaching method (Walker, 2014), professional development (Lorimer, 2016), and student art activities (Thomas, 2009; Pitri, 2013), these articles were not connected to an intentional research design. Research designs included case study (James, 1997; Karademir, 2016) and ethnography (Guay, 2000; Petsch, 2000; Mars, 2016). Some studies attempted to tie general pedagogy to teaching for creativity in specific subjects. For example, Sullivan (2011) and Meyer and Lederman (2013) mapped science instruction to the demonstration of creativity, and Donovan et al. (2014) mapped creativity to technology. Conversely, other studies used an inductive framework. Robson and Rowe (2012) first identified child-initiated activities, which were then interpreted as supportive of creative thinking (p. 355). Walmsley (2013) focused on observing a co-creation process using theater, characterizing by “creative energy” and “rawness” (p. 114).

### Data collection and analysis methods

Several articles omitted citations on the process for collecting observation data. As a result, this obscured the researchers' process for data saturation to ensure a thorough understanding of the creative process. One article used critical incident sampling by selecting members for classroom observation based on reporting in surveys or interviews (Meyer and Lederman, 2013). Other studies observed students identified as gifted (e.g., Karademir, 2016).

Researchers used “thick description” (Geertz, 1973) to provide detailed description of the observation site and activities and thus demonstrate authenticity and validity of data. Some articles also used triangulation—the integration of observation and interview data to corroborate themes and establish validity across data sources (Waite, 2014). Thick description of the creative process included examples of modeling in art instruction (Budge, 2016), online music collaboration (Biasutti, 2015) and student-teacher interactions (James, 1997; Thomas, 2009). Photographs of student drawings and creative projects (Pitri, 2013; Walker, 2014; Vuk et al., 2015; Karademir, 2016; Lorimer, 2016) also strengthened observation descriptions.

Qualitative arts-based researchers build relationships with artists and students when observing the artistic process (Bresler, 2008). Accordingly, researchers discuss how empathy can deepen understanding and counteract bias (Bogdan and Biklen, 2007) to enhance validity. Most studies, though, did not disclose the *type* of observer role selected (e.g., Walker, 2014); a few specified the *participant* type (Guay, 2000; Petsch, 2000; Budge, 2016; Mars, 2016), and others described researcher involvement to understand student work in the classroom (James, 1997; Thomas, 2009; Pitri, 2013; Donovan et al., 2014; Lorimer, 2016).

Data analysis procedures varied, often using qualitative observations to triangulate—that is, confirm or disconfirm other data sources. In some articles, interview and survey results overshadowed observation data (Bertling, 2015; Lorimer, 2016) making it difficult to parse out the contributions that observation data made to understanding the nature of creativity (Godart and Mears, 2009; Watson, 2014). Many analytic coding procedures were not supported by methodological citations. Even when specified, the preparation of field notes for analysis and the relation of discrete codes to final themes remained vague. Examples of well-articulated coding of observations included the constant comparative method (Meyer and Lederman, 2013), content analysis method (Karademir, 2016), interaction analysis (Sullivan, 2011), semantic analysis (Thomas, 2009) and symbolic interactionism (James, 1997).

## Conclusion

This review shows that observation methods are woefully underutilized and, generally, lack methodological rigor. It's imperative for researchers to uphold rigorous research standards in the area of observational studies of creativity in learning, just as they would when using creativity measures, for example. The varied quality of observation studies in this review highlights the need for clearer and stronger observation methods to advance the understanding of creativity processes in learning.

The lack of definitional clarity emphasized by Plucker et al. (2004) more than a decade ago remains a critical need in observation studies. Researchers in both qualitative and quantitative traditions must clarify how they define, operationalize, and observe creativity. We found that the majority of observation studies lacked conceptual specificity, short-changing their potential contributions to the field. Students should be encouraged to use observation of teaching and learning creativity as an empirical method in research courses and dissertation work, but they must be equipped with the methodological skill and conceptual rigor to link creative development to specific instructional and environmental factors. Strong student research will help build a pipeline of new researchers observing creativity in a broader variety of domains.

The dominance of product-only assessments of creativity in research explains in part why the creativity field struggles to develop and scale curricular and instructional supports that promote the creative process in everyday teaching and learning in Cho et al. (2013). As we have described, several diverse exemplars in the creativity literature demonstrate how rigorous observation studies can help produce richer and clearer accounts of creativity in educational settings. Without continued investment, observation in creativity research may remain an underdeveloped method. Furthermore, instructional and environmental progress in support of creative development of students will lag behind our ambitions as a field.

## Author contributions

JK-B developed the concept of the review, searched for the articles and reviewed the articles, wrote the intro, conclusion and the qualitative review. RA helped develop the concept and reviewed the quantitative articles and wrote the quantitative review and added to the table.

### Conflict of interest statement

The authors declare that the research was conducted in the absence of any commercial or financial relationships that could be construed as a potential conflict of interest.
